# Supplementary material for: Active T1 transitions in cellular networks
Source: Eur Phys J E Soft Matter. 2022 Mar 23;45(3):29. doi: 10.1140/epje/s10189-022-00175-5 (PMC8942949; doi:10.1140/epje/s10189-022-00175-5)
Supplement: Supplementary file 5 — (pdf 378 KB) [file 10189_2022_175_MOESM5_ESM.pdf]

Supplemental material:
Active T1 transitions in cellular networks

Charlie Duclut,[1,*] Joris Paijmans,[1,*] Mandar M. Inamdar,[2]
Carl D. Modes,[3,4,5] and Frank Jülicher[1,4,5]

[1] *Max Planck Institute for the Physics of Complex Systems, Nöthnitzer Str. 8, 01187 Dresden, Germany*

[2] *Department of Civil Engineering, Indian Institute of Technology Bombay, Powai, Mumbai 400076, India*

[3] *Max Planck Institute for Molecular Cell Biology and Genetics (MPI-CBG), Dresden 01307, Germany*

[4] *Center for Systems Biology Dresden, Pfotenhauerstrasse 108, 01307 Dresden, Germany*

[5] *Cluster of Excellence, Physics of Life, TU Dresden, Dresden 01307, Germany*

[*] These authors contributed equally.

Appendix A: Vertex model simulations

In this appendix we provide a more detailed description of the vertex model simulations.

1. Dimensionless parameters

In our simulations of the vertex model we use parameters expressed in dimensionless units. To this end, we will choose τ_Λ as the typical timescale, $A_0^{1/2}$ as the typical length scale and KA_0^2 as the typical energy scale in our model. The effective parameters are thus reduced to $\bar{\Lambda}_{mn} = \Lambda_0/(KA_0^{3/2})$, $\bar{\Gamma} = \Gamma/(KA_0)$, $\Delta\bar{\Lambda} = \Delta\Lambda/(KA_0^{3/2})$. The parameters τ_Λ, K, and A_0 are unity in these units. Throughout the manuscript, we use dimensionless units and omit the bar on the parameter symbols for simplicity. We choose the parameter values $\Lambda_0 = 0.12$ and $\Gamma = 0.04$ which are known to produce network configurations that agree well with those observed in the wing disk epithelium [1]. Other parameter values are listed in Table S1.

2. Model initialization and simulation

Each simulation is initialized as a network of $N_x = 20$ by $N_y = 20$ regular hexagonal cells, with box dimensions L_x and L_y set such that the network work function (1) is in its ground state [1]. We first propagate the system under a fixed boundary condition and without anisotropy ($\beta_0 = \Sigma_0^{\mathrm{a}} = 0$),

vertex model parameters			
**symbol**	**explanation**	**value**	**unit**
mechanics			
Λ_0	mean line tension	0.12	$KA_0^{3/2}$
Γ	perimeter elasticity	0.04	KA_0
β_0	steady-state line tension anisotropy	0.50	-
Σ_0^{a}	steady-state active cell stress magnitude	0.04	KA_0
T_{a}	anisotropy activation time	1	τ_Λ
dynamics			
$\Delta\Lambda$	line tension fluctuations magnitude	0.06	$KA_0^{3/2}$
δt	time step for numerical integration	0.01	τ_Λ

TABLE S1. Parameter values used in the simulations of the vertex model, expressed in dimensionless units. In case different values are used for simulation results, it is stated in the caption of the figure showing the results.

until the system has reached a steady state configuration at $t = 50\tau_\Lambda$. Once the system is prepared, we distinguish the following two cases.

(i) *Fixed box boundary condition.* In this case, after reaching an isotropic steady state, the fixed box boundary condition is kept and one of the two anisotropic contributions is included. In the case of an instantaneous activation, this contribution is included in the work function immediately at the end of the equilibration time. In the case of a gradual activation, the anisotropic contribution is added with an exponential increase $1 - \mathrm{e}^{-t/T_\mathrm{a}}$. The system is then propagated for a time $t = 15\tau_\Lambda$. This procedure is repeated $N = 100$ times and the corresponding simulations results are displayed in Fig. 2 for the gradual activation case and in Fig. S2 for the instantaneous activation case.

(ii) *Stress-free boundary condition.* In this case, after reaching an isotropic steady state under a fixed box boundary condition, one of the two anisotropic contributions is included and the box is left free to deform such that no stress is exerted on the simulation box. Similarly to the fixed box case, gradual and instantaneous activations are considered. The system is propagated for a time $t = 10\tau_\Lambda$. This procedure is repeated $N = 100$ times and the corresponding simulations results are displayed in Fig. 3 for the gradual activation case and in Fig. S1 for the instantaneous activation case.

3. T1 transitions

In this implementation of the vertex model, a full T1 transition is divided into 2 steps. First, two connected three-fold vertices that reach a distance $\mathcal{L}_{mn}$ lower than a certain threshold value, will merge to form a four-fold vertex. Next, a tentative split into two vertices is attempted in both possible topologies of the network. If in one of the topologies the two vertices are pulled apart by a force, the four-fold vertex is unstable. If the four-fold vertex is unstable for both topologies, the topology that maximizes the force magnitude is chosen. In the case where the four-fold vertex is stable, it is kept as such and a new tentative split is attempted at the next time step. In case that cell neighbors have changed as compared to before the merger of the vertices, we call it a full T1 transition. Upon a T1 transition, which creates of a new bond at time t_0, the initial value of the bond tension $\Lambda_{mn}(t_0)$ is drawn from a normal distribution with mean $\bar{\Lambda}_{mn}$ and variance $\Delta\Lambda$. After the new bond is created, the system is again relaxed to a force-balanced state.

4. Constant cell number ensemble

In all the vertex model simulations in this paper, we use a fixed cell number ensemble. In some rare cases, bond tension fluctuations can drive the area of a cell below a critical area. Below this critical area, the cell area imposed by the local minimum of this cell's work function is zero. The cell would therefore shrink to have an area that is zero. When the cell area reaches a value below a certain set threshold, the cell is extruded from the network and replaced by a vertex which has the same order as the neighbor number of the removed cell. In order to keep the number of cells in the tissue constant, a randomly chosen cell divides.

In case a cell divides, a new bond is created running through the cell center. The new bond makes an angle with the x-axis which is drawn from a uniform distribution between 0 and π. The two daughter cells have the same preferred area as the mother cell, simplifying the more realistic case of the continuous growth of the cell area. After each cell division, the configuration of the cell network is changed in order to minimize the work function.

Appendix B: Notation and definitions

1. Velocity gradient tensor

The motion of cells in the tissue is described by the coarse-grained cell velocity field v_j (or V_j for the vertex model). Deformations of the network are proportional to gradients in this velocity field $v_{ij} = \partial_i v_j$, where v_{ij} is the velocity gradient tensor. The trace of this tensor, v_{kk} (summation over repeated Cartesian indices is implied), corresponds to local isotropic growth of the tissue. The traceless-symmetric part of the velocity gradient tensor, denoted $\tilde{v}_{ij}$, corresponds to anisotropic deformations, and its antisymmetric part, the vorticity tensor ω_{ij}, characterizes local rotations. The velocity gradient tensor can thus be decomposed as:

$$v_{ij} = \frac{1}{2} v_{kk}\delta_{ij} + \tilde{v}_{ij} + \omega_{ij}, \tag{B1}$$

where in two dimensions we have $\omega_{ij} = -\omega\varepsilon_{ij}$ with ε_{ij} the generator of counterclockwise rotation with $\varepsilon_{xy} = -1$, $\varepsilon_{yx} = 1$ and $\varepsilon_{xx} = \varepsilon_{yy} = 0$.

2. Stress tensor

Similarly to the velocity gradient tensor, the tissue stress tensor σ_{ij} can also be decomposed into

$$\sigma_{ij} = \frac{\sigma_{kk}}{d}\delta_{ij} + \tilde{\sigma}_{ij} \, . \tag{B2}$$

where d is the spatial dimension and $\tilde{\sigma}_{ij}$ is the shear stress. Note that in the absence of chiral terms the stress tensor is symmetric, and we have therefore not included the antisymmetric contribution to the previous equation.

3. Corotational time derivative of tensors

The corotational time derivative of a tensor M_{ij} is defined as:

$$\frac{\mathrm{D}M_{ij}}{\mathrm{D}t} = \frac{\mathrm{d}M_{ij}}{\mathrm{d}t} + \omega_{ik}M_{kj} + \omega_{jl}M_{il} . \tag{B3}$$

where ω_{ij} is the vorticity tensor of the fluid.

4. Nematic tensors in two dimensions

In dimension two, a traceless symmetric tensor M_{ij} (that we call nematic tensor) has two degrees of freedom and can be written in terms of its Cartesian coordinates as:

$$\boldsymbol{M} = \begin{pmatrix} M_{xx} & M_{xy} \\ M_{xy} & -M_{xx} \end{pmatrix} , \tag{B4}$$

or it can equivalently be decomposed into a norm $M = |\boldsymbol{M}|$ and angle Θ as:

$$\boldsymbol{M} = M \begin{pmatrix} \cos(2\Theta) & \sin(2\Theta) \\ \sin(2\Theta) & -\cos(2\Theta) \end{pmatrix} , \tag{B5}$$

with $M = \sqrt{M_{xx}^2 + M_{xy}^2}$ and $\Theta = \frac{1}{2}\arctan(M_{xy}, M_{xx})$, where the function arctan gives the arc tangent of M_{xy}/M_{xx}, taking into account in which quadrant the point (M_{xy}, M_{xx}) lies. From Eq. (B5), one directly sees that $\boldsymbol{M}^2 = M^2\mathbb{1}$ for a nematic tensor in two dimensions.

Appendix C: Instantaneous activation of the anisotropy

In addition to the gradual exponential activation of the anisotropy that we have discussed in the main text, we have also considered an instantaneous activation. In this case, after equilibrating the

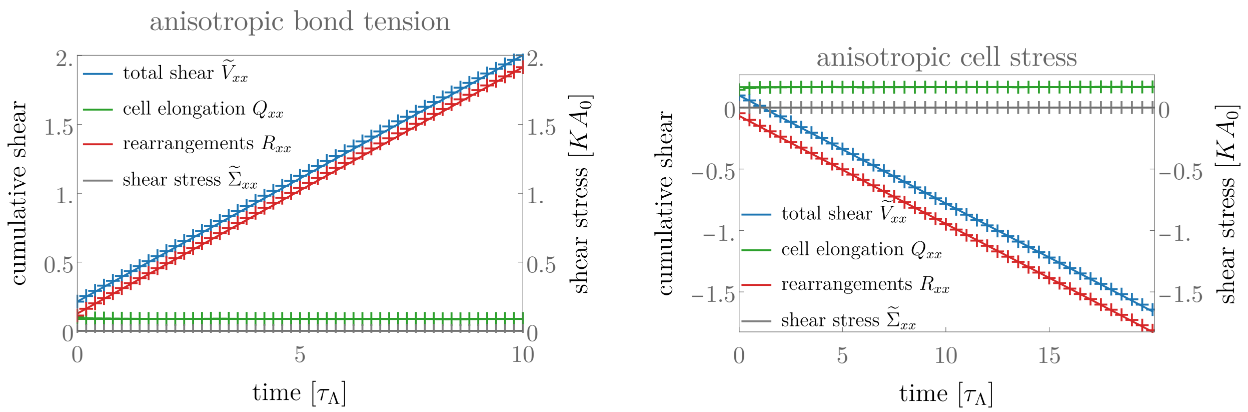

FIG. S1. Anisotropic vertex model simulations with stress-free boundary conditions and instantaneous activation of the anisotropic bond tension (left) or anisotropic cell stress (right). Crosses are obtained by averaging 100 realizations of the vertex model simulations (error bars are smaller than the marker size); solid lines are obtained by fitting the hydrodynamic model (see App. D for details and values of the fitted parameters).

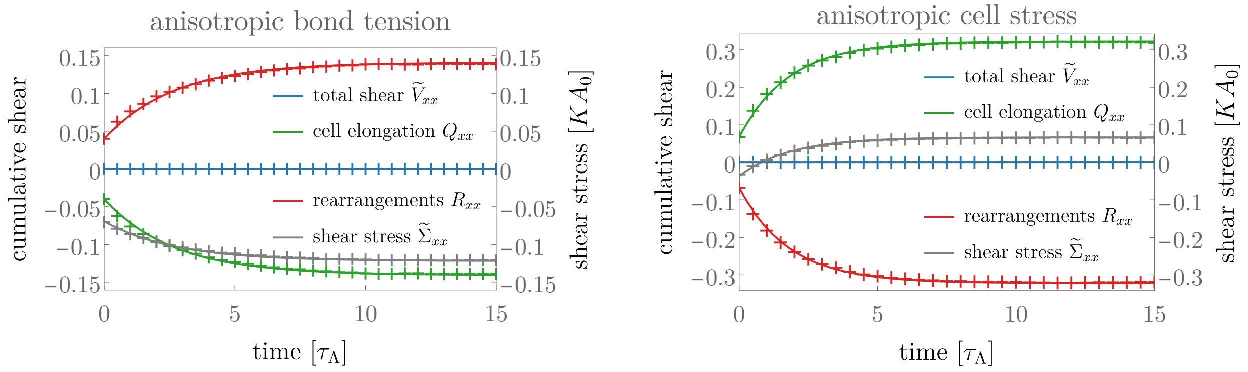

FIG. S2. Anisotropic vertex model simulations with fixed box boundary conditions and instantaneous activation of the anisotropic bond tension (left) or anisotropic cell stress (right). Crosses are obtained by averaging 100 realizations of the vertex model simulations (error bars are smaller than the marker size); solid lines are obtained by fitting the hydrodynamic model (see App. D for details and values of the fitted parameters).

system with isotropic properties for a time $t = 50\tau_\Lambda$, the anisotropic cell stress or the anisotropic bond tension are immediately set to their steady-state values. An exponential relaxation of cell elongation to its steady-state value is observed and fitted to the continuum model (see App. D for details on the fitting procedure). We display in Fig. S1 the results of the simulations and fits in the case of stress-free boundary conditions, and in Fig. S2 the case of a fixed box boundary condition.

Appendix D: Continuum model for anisotropic tissues

Here, we discuss how the continuum model is used to fit the vertex model data. We recall for convenience the shear decomposition and the constitutive equations for the tissue stress and rate of cellular rearrangements:

$$\tilde{v}_{ij} = \frac{\mathrm{D}q_{ij}}{\mathrm{D}t} + r_{ij} \, , \tag{D1a}$$

$$r_{ij} = \frac{1}{\tau} q_{ij} + \lambda(t) p_{ij} \, , \tag{D1b}$$

$$\tilde{\sigma}_{ij} = \mu q_{ij} + \zeta(t) p_{ij} \, . \tag{D1c}$$

The dynamics and steady states depend crucially on the imposed boundary conditions, and we discuss below the stress-free and fixed box boundary conditions studied in the main text.

1. Fixed box boundary condition

For a fixed box boundary condition, the tissue cannot deform and $\tilde{v}_{ij} = 0$, such that shear decomposition (D1a) reduces to:

$$q'_{ij}(t) = -r_{ij}(t) \, , \tag{D2}$$

where the prime denotes the time derivative.

a. Steady state

At steady state, cell elongation is constant, and we deduce from Eq. (D2) that $r_{ij} = 0$. We thus obtain:

$$q^{\mathrm{ss}}_{ij} = -\lambda_0 \tau p_{ij} \, , \quad \tilde{\sigma}^{\mathrm{ss}}_{ij} = \left(-\mu\tau\lambda_0 + \zeta_0\right) p_{ij} = -\mu\tau\tilde{\lambda}_0 p_{ij} \, , \tag{D3}$$

where λ_0 and ζ_0 are the steady-state values of $\lambda(t)$ and $\zeta(t)$, respectively. We have also introduced $\tilde{\lambda}_0 = \lambda_0 - \zeta_0/(\mu\tau)$. The coefficients $-\lambda_0\tau$ and $-\mu\tau\tilde{\lambda}_0$ are then obtained from the vertex model data by computing the steady-state mean cell elongation and the steady-state tissue shear stress, respectively.

Note that in the main text we only discuss the steady-states values λ_0, ζ_0, and $\tilde{\lambda}_0$ of the parameters, and we have dropped their subscript 0 for simplicity.

b. *Instantaneous activation*

We consider instantaneous activation of the anisotropy in the vertex model. We therefore consider $\lambda(t) = \lambda_0 \Theta(t)$ and $\zeta(t) = \zeta_0 \Theta(t)$ where $\Theta(t < 0) = 0$ and $\Theta(t \geq 0) = 1$ in the continuum description. In this case, Eq. (D1a) can be solved and yields:

$$q_{ij}(t) = q_{ij}(t = 0^+)\mathrm{e}^{-t/\tau} - \lambda_0 \tau \left(1 - \mathrm{e}^{-t/\tau}\right) p_{ij} \,, \tag{D4}$$

where $q_{ij}(t = 0^+)$ is the value of cell elongation immediately after activation of the anisotropy. The exponential relaxation given by Eq. (D4) can be fitted to the vertex model data for cell elongation to obtain the timescale τ.

From this fit and the values obtained from steady state, the only remaining parameter is μ, which is obtained from fitting the continuum model shear stress exponential relaxation

$$\tilde{\sigma}_{ij}(t) = \mu q_{ij}(t = 0^+)\mathrm{e}^{-t/\tau} + \left[\zeta_0 - \lambda_0 \mu \tau \left(1 - \mathrm{e}^{-t/\tau}\right)\right] p_{ij} \,, \tag{D5}$$

to the tissue shear stress from the vertex model data.

c. *Gradual activation*

We consider an exponential gradual adaptation of the anisotropy in the vertex model. In the continuum description, we introduce two adaptation times τ_λ and τ_ζ, such that $\lambda(t) = \lambda_0(1 - \mathrm{e}^{-t/\tau_\lambda})$ and $\zeta(t) = \zeta_0(1 - \mathrm{e}^{-t/\tau_\zeta})$. Solving Eq. (D1a) with $q_{ij}(0) = 0$, we obtain:

$$q_{ij}(t) = -\lambda_0 \tau p_{ij} \left(1 + \frac{\tau \mathrm{e}^{-t/\tau} + \tau_\lambda \mathrm{e}^{-t/\tau_\lambda}}{\tau - \tau_\lambda}\right) . \tag{D6}$$

This solution is fitted against the data from the vertex model for cell elongation to obtain τ_λ and τ. Using these fitted values and the parameter values obtained from steady state, the remaining parameters to obtain are μ and τ_ζ. They are obtained from fitting the continuum model shear stress

$$\tilde{\sigma}_{ij}(t) = \left[\zeta_0 \left(1 - \mathrm{e}^{-t/\tau_\zeta}\right) - \lambda_0 \mu \tau \left(1 + \frac{\tau \mathrm{e}^{-t/\tau} + \tau_\lambda \mathrm{e}^{-t/\tau_\lambda}}{\tau - \tau_\lambda}\right)\right] p_{ij} \,, \tag{D7}$$

to the tissue shear stress from the vertex model simulations. Note that the fits displayed in Figs. 2 and 3 of the main text have been obtained using a single activation timescale $\tau_a = \tau_\lambda = \tau_\zeta$.

2. Stress-free boundary condition

Under a stress-free boundary condition, we impose the total stress σ_{ij} to vanish. The tissue is left free to deform and the tissue deformation tensor v_{ij} is unconstrained. A vanishing stress implies directly from Eq. (D1c) that cell elongation and the anisotropic stress are proportional:

$$q_{ij}(t) = -\frac{\zeta(t)}{\mu} p_{ij} \,. \tag{D8}$$

a. *Steady state*

At steady state, we obtain for stress-free boundary conditions:

$$q_{ij}^{\rm ss} = -\frac{\zeta_0}{\mu} p_{ij} \,, \quad \tilde{v}_{ij}^{\rm ss} = \tilde{\lambda}_0 p_{ij} \,, \tag{D9}$$

where $\tilde{\lambda}_0 = \lambda_0 - \zeta_0/(\mu\tau)$ and where ζ_0 are the steady-state values of $\lambda(t)$ and $\zeta(t)$, respectively. The coefficients $-\zeta_0/\mu$ and $\tilde{\lambda}_0$ are then obtained from the vertex model data by computing the steady-state mean cell elongation and the steady-state tissue shear rate, respectively.

Note that in the main text we only discuss the steady-states values λ_0, ζ_0, and $\tilde{\lambda}_0$ of the parameters, and we have dropped their subscript 0 for simplicity.

b. *Instantaneous activation*

We consider instantaneous activation of the anisotropy in the vertex model. We therefore consider $\lambda(t) = \lambda_0 \Theta(t)$ and $\zeta(t) = \zeta_0 \Theta(t)$ where $\Theta(t < 0) = 0$ and $\Theta(t \geq 0) = 1$ in the continuum description. In this case and for stress-free boundary conditions, we remark that Eq. (D8) implies $\tilde{v}_{ij} = r_{ij}$, and the instant adaptation case can be fitted using only the parameter values extracted from the steady-state analysis.

c. *Gradual activation*

We consider an exponential gradual adaptation of the anisotropy in the vertex model. In the continuum description, we introduce two adaptation times τ_λ and τ_ζ, such that $\lambda(t) = \lambda_0(1-{\rm e}^{-t/\tau_\lambda})$ and $\zeta(t) = \zeta_0(1 - {\rm e}^{-t/\tau_\zeta})$. In this case, Eq. (D1c) reads:

$$q_{ij}(t) = -\frac{\zeta_0}{\mu}\left(1 - {\rm e}^{-t/\tau_\zeta}\right) p_{ij} \,. \tag{D10}$$

This equation can be fitted to the cell elongation data to obtain the timescale τ_ζ. The second activation timescale τ_λ as well as τ can then be obtained by fitting the continuum model shear rate

$$v_{ij}(t) = \left[\tilde{\lambda}_0 - \left(\frac{\zeta_0}{\mu\tau} + \tilde{\lambda}_0\right) \mathrm{e}^{-t/\tau_\lambda} + \frac{\zeta_0}{\mu}\left(\frac{1}{\tau} - \frac{1}{\tau_\zeta}\right) \mathrm{e}^{-t/\tau_\zeta}\right] p_{ij} \tag{D11}$$

to the vertex model shear rate. Note that the fits displayed in Figs. 2 and 3 of the main text have been obtained using a single activation timescale $\tau_{\mathrm{a}} = \tau_\lambda = \tau_\zeta$.

3. Parameter values obtained from the fits

In Table S2, we display the parameters obtained from fitting the continuum model to the vertex model simulations. Note that for the stress-free boundary condition, only the reduced parameters ζ_0/μ and $\tilde{\lambda}_0 = \lambda_0 - \zeta_0/(\mu\tau)$ can be obtained.

For the anisotropic cell stress case, the agreement between the four realizations of the model (fixed box or stress-free boundary conditions, and gradual or instantaneous activation) is excellent, and the discrepancy between fitted values is minimal. For the anisotropic bond tension case, we however observe relatively strong discrepancies between the different fits between the different activation procedures and boundary conditions. This is likely due to nonlinear properties of the vertex models, not captured by our linear version. Indeed, these nonlinear properties have been shown to occur, for the same vertex model parameters as those studied here, at relatively large shear rate of order $\tilde{v}_{xx} \sim 0.2$ [2]. In the anisotropic model that we consider here, the system is not sheared by an externally-imposed shear rate, but the shearing is rather induced by the anisotropic activity. In the stress-free boundary condition (see Figs. 3 and S1), we can compute this induced shear rate and find that it is $\tilde{v}_{xx} \simeq 0.18$ for our choice of parameters and for anisotropic bond tension, and $\tilde{v}_{xx} \simeq -0.089$ for anisotropic cell stress. The anisotropic cell stress version is therefore driven at a lower shear rate and is thus better captured by the linear model. A nonlinear version of the model, constructed in the spirit of what has been done in Ref. [2], is beyond the scope of this work.

Appendix E: Supplemental movies

1. Movie 1: anisotropic bond tension with fixed boundary conditions

Description: Snapshots of the dynamics of the vertex model with fixed box boundary condition and anisotropic bond tension. Each cell is colored according to the norm of its elongation tensor

		Parameter values obtained from fit									
		**Fixed box**							**Stress-free**		
		μ	τ	λ_0	ζ_0	ζ_0/μ	$\tilde{\lambda}_0$	τ_a	ζ_0/μ	$\tilde{\lambda}_0$	τ_a
**anisotropic**	gradual activation	0.64	3.0	-0.047	0.031	0.048	-0.063	0.068	0.087	-0.18	0.41
**bond tension**	instant. activation	0.52	2.7	-0.052	0.048	0.093	-0.087	/	0.087	-0.18	/
**anisotropic**	gradual activation	0.42	1.9	0.17	0.069	0.16	0.085	1.2	0.17	0.089	1.2
**cell stress**	instant. activation	0.41	1.8	0.18	0.064	0.16	0.090	/	0.17	0.088	/

TABLE S2. Parameter values for the hydrodynamic model with anisotropy, obtained by fitting Eqs. (D1a) to (D1c) to the vertex model simulation data. Details of the fitting procedure can be found in App. D. Note that in the main text we have dropped the subscript 0 of λ_0, ζ_0, and $\tilde{\lambda}_0$ for simplicity.

and the purple bar in its center indicates the axis of elongation. When the color of the surrounding box becomes red, the mean bond tension $\bar{\Lambda}_{mn}(t)$ of each bond $\langle mn\rangle$ in the system is gradually activated with the following time-dependence:

$$\bar{\Lambda}_{mn}(t) = \bar{\Lambda}^0 + \bar{\Lambda}^0\beta_0\left(1 - e^{-t/T_a}\right)\boldsymbol{\mathcal{P}} : \hat{\mathbf{H}}_{mn}\,, \tag{E1}$$

where $\hat{\mathbf{H}}_{mn}$ is the unit nematic tensor of the bond between vertices m and n, and the nematic tensor $\boldsymbol{\mathcal{P}}$ is along the y-axis in this movie. As a consequence, bond tension is higher for bonds which are more aligned with the vertical (y) direction.

Parameters: $\Lambda_0 = 0.12$, $\Delta\Lambda = 0.06$, $\Gamma = 0.04$, $\beta_0 = 0.5$, $\Sigma_0^a = 0$, $T_a = 1$, $\delta t = 0.01$.

2. Movie 2: anisotropic bond tension with stress-free boundary conditions

Description: Snapshots of the dynamics of the vertex model with stress-free boundary condition and anisotropic bond tension. Each cell is colored according to the norm of its elongation tensor, and the purple bar in its center indicates the axis of elongation. When the color of the surrounding box becomes red, the mean bond tension $\bar{\Lambda}_{mn}(t)$ of each bond $\langle mn\rangle$ in the system is gradually activated. As a consequence, the higher mean bond tension along the y axis, active T1 transitions are triggered and the box stretches along the x direction.

Parameters: $\Lambda_0 = 0.12$, $\Delta\Lambda = 0.06$, $\Gamma = 0.04$, $\beta_0 = 0.5$, $\Sigma_0^a = 0$, $T_a = 1$, $\delta t = 0.01$.

3. Movie 3: anisotropic cell stress with fixed boundary conditions

Description: Snapshots of the dynamics of the vertex model with fixed box boundary condition and anisotropic cell stress. Each cell is colored according to the norm of its elongation tensor, and

the purple bar in its center indicates the axis of elongation. When the color of the surrounding box becomes red, the anisotropic stress contribution $\Sigma_{ij}(t)$ of the vertex model work function is gradually activated with the following time-dependence:

$$\Sigma_{ij}(t) = \Sigma_0^{\mathrm{a}} \left(1 - \mathrm{e}^{-t/T_{\mathrm{a}}}\right) \mathcal{P}_{ij}\,, \tag{E2}$$

where the nematic tensor $\mathcal{P}_{ij}$ is along the y-axis in this movie. Note that cells are more contractile along $\mathcal{P}_{ij}$ and therefore elongate in the direction normal to this axis.

Parameters: $\Lambda_0 = 0.12$, $\Delta\Lambda = 0.06$, $\Gamma = 0.04$, $\beta_0 = 0$, $\Sigma_0^{\mathrm{a}} = 0.04$, $T_{\mathrm{a}} = 1$, $\delta t = 0.01$.

4. Movie 4: anisotropic cell stress with stress-free boundary conditions

Description: *Left panel.* Snapshots of the dynamics of the vertex model with stress-free boundary condition and anisotropic cell stress. Each cell is colored according to the norm of its elongation tensor and the purple bar in its center indicates the axis of elongation. When the color of the surrounding box becomes red, the mean bond tension $\bar{\Lambda}_{mn}(t)$ of each bond $\langle mn \rangle$ in the system is gradually activated. As a consequence of cell elongation, T1 transitions are triggered and the box stretches along the y direction. *Right panel.* Cumulative shear decomposition of the simulation displayed on the left panel. The color code of the decomposition is the following. Blue represents the total shear, green the contribution from cell elongation to shear, red the contribution of T1 transitions, and purple the contribution due to correlations.

Parameters: $\Lambda_0 = 0.12$, $\Delta\Lambda = 0.06$, $\Gamma = 0.04$, $\beta_0 = 0$, $\Sigma_0^{\mathrm{a}} = 0.04$, $T_{\mathrm{a}} = 1$, $\delta t = 0.01$.

[1] R. Farhadifar, J.-C. Röper, B. Aigouy, S. Eaton, and F. Jülicher, Curr. Biol. **17**, 2095 (2007).

[2] C. Duclut, J. Paijmans, M. M. Inamdar, C. D. Modes, and F. Jülicher, Cells & Development , 203746 (2021).
